# Alcohol‐cancer risk communication on social media: A content analysis of alcohol‐related Instagram and TikTok posts

**DOI:** 10.1111/acer.70260

**Published:** 2026-02-22

**Authors:** Joseph Alejandro, Christina Mair, Robert W. S. Coulter, Kar‐Hai Chu, Alexa Pierce, Kennedy Sawicki, Arpita Tripathi, Jaime E. Sidani

**Affiliations:** ^1^ Department of Behavioral and Community Health Sciences, School of Public Health University of Pittsburgh Pittsburgh Pennsylvania USA; ^2^ Center for Social Dynamics and Community Health, School of Public Health University of Pittsburgh Pittsburgh Pennsylvania USA; ^3^ Department of Health Policy and Management, School of Public Health University of Pittsburgh Pittsburgh Pennsylvania USA; ^4^ Department of Human Genetics, School of Public Health University of Pittsburgh Pittsburgh Pennsylvania USA

**Keywords:** alcohol, cancer, Instagram, social media, TikTok

## Abstract

**Background:**

Despite consistent evidence of the relationship between alcohol use and cancer risk, awareness of the alcohol‐cancer link remains low among American adults. Given the increasing prominence of social media as a health information source, this study systematically examined samples of alcohol‐related Instagram and TikTok posts to identify mentions of the alcohol‐cancer link and characterize post content.

**Methods:**

We collected public Instagram and TikTok posts made on or before May 12, 2024, via a hashtag‐based search for posts tagged with #alcohol, #beer, #wine, #liquor, and #cocktail. Following an iterative codebook development process, two independent coders qualitatively reviewed random 2% (*n* = 1666) samples of Instagram posts and 20% (*n* = 725) samples of TikTok posts. Cohen's *κ* for each coding category ranged from 0.65 to 1.00 for Instagram posts and 0.61 to 1.00 for TikTok posts, while percent agreement for codes with low training sample prevalence ranged from 96.0% to 100% for both platforms. We examined posts identified as featuring intoxication or binge or high‐intensity drinking, having been made by an influencer, featuring alcohol products, and targeting specific groups using inductive thematic content analysis.

**Results:**

Across both platforms, only one post, a TikTok video, mentioned the relationship between alcohol use and increased cancer risk. Posts often positively depicted alcohol and commonly considered beverages containing large amounts of alcohol as single drinks. 48.6% of Instagram posts were made by commercial entities and 69.7% showed an alcohol product. 68.1% of TikTok posts were posted by influencers, 52.7% were comedic, and 24.7% referenced intoxication, binge, or high‐intensity drinking.

**Conclusions:**

Cancer risk communication is largely absent among Instagram and TikTok posts, which often portray alcohol use in a positive light. Social media represents a critical, yet underutilized, channel for disseminating public health information about the alcohol‐cancer link. Content analyses can inform strategic, audience‐tailored messaging for improving public awareness.

## Graphical abstract

This study examined alcohol‐related Instagram and TikTok posts to assess the prevalence of alcohol‐cancer risk communication and characterize post content. Posts often positively depicted alcohol and, of 2391 posts across both platforms, only one TikTok video mentioned the relationship between alcohol use and cancer risk.

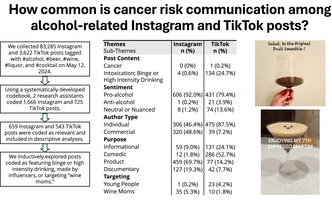

## INTRODUCTION

Alcohol use is responsible for approximately 4.1% of new cancer cases worldwide and 5.4% of new cancer cases in the United States (Islami et al., 2024; Rumgay et al., 2021). A leading modifiable cancer risk factor, alcohol consumption is associated with an increased risk for cancers of the head and neck, esophagus, liver, female breast, and colorectum, among others (Bagnardi et al., 2015). First linked to increased cancer risk in the early twentieth century, recent studies continue to highlight the carcinogenic effects of alcohol consumption at any level (International Agency for Research on Cancer Working Group on the Evaluation of Carcinogenic Risks to Humans, 1988). For example, a recent meta‐analysis by Sohi et al. (2024) found that, compared to nondrinking women, women consuming a single drink per day had a 10% increased risk for developing breast cancer. Similar or stronger associations have been documented between the consumption of approximately one to two drinks per day and esophageal (83% increased risk), laryngeal (63% increased risk), and colorectal cancers (9% increased risk), each exhibiting a dose–response relationship at even low levels of drinking (Jun et al., 2023). Identified as a public health priority, reducing alcohol consumption to current United States dietary guidelines (i.e., ≤2 drinks per day for men or ≤1 drink per day for women) could prevent close to 17,000 cancer deaths per year among American adults (Esser et al., 2024; Office of the Surgeon General, 2025).

Despite the growing body of evidence documenting that alcohol consumption is associated with increased cancer risk, public awareness of the link between alcohol and cancer is low. Recent studies suggest that approximately two‐thirds of Americans are unaware of the association between alcohol and cancer risk (Kiviniemi et al., 2021; Seidenberg et al., 2023), with the knowledge of the relationship between alcohol and breast cancer exceptionally low (Khushalani et al., 2020). While trending downward in recent years, 8% of Americans consider moderate drinking to have beneficial health effects, likely due to the lingering impacts of prior messaging suggesting health benefits associated with lower levels of alcohol consumption (e.g., “a glass of red wine a day”) since proven untrue (Anderson et al., 2023; Brenan, 2024). Beliefs that drinking beer, wine, or liquor decreases or has no impact on cancer risk are associated with 72%, 59%, and 38% increased odds, respectively, for current alcohol consumption. Therefore, increasing public awareness of alcohol's carcinogenic effects presents an opportunity to reduce alcohol use and the subsequent incidence of alcohol‐related cancers in the United States (Gapstur et al., 2022; Rohde et al., 2023).

Popular social media platforms, such as Instagram and TikTok, are increasingly present in the lives of American adults, many of whom turn to social media for health‐related information (Gottfried, 2024; Lama et al., 2022). The content that people encounter on social media may influence their attitudes toward or awareness of health‐related topics such as the relationship between alcohol and cancer risk and in turn affect their health behaviors (Chen & Wang, 2021). However, the presence of information about the alcohol cancer link on social media has thus far been limited to an examination of Twitter posts (King et al., 2023). Research further suggests that people with higher exposures to alcohol‐related social media content are more likely to drink, thereby increasing their risk for developing alcohol‐associated cancers (Curtis et al., 2018). Given their popularity and the potential role of Instagram and TikTok in shaping public awareness of the alcohol‐cancer link, with additional concerns specific to alcohol‐related content, we sought to answer the following research question: What is the prevalence of cancer‐risk communication among alcohol‐related Instagram and TikTok posts? To do so, we systematically examined alcohol‐related Instagram and TikTok posts to identify those referencing the association between alcohol and cancer risk. We also broadly characterized post content to inform future content analyses and risk communication efforts.

## MATERIALS AND METHODS

### Data collection

We used web scraping tools from Apify, a third‐party data extraction platform, to collect publicly available Instagram and TikTok posts tagged with one or more of the hashtags #alcohol, #beer, #wine, #liquor, and #cocktail. In addition to their overall popularity, these platforms are varied in terms of their user demographics, with Instagram, for example, being more popular among American adults ages 30–49 (Gottfried, 2024). We selected our data extraction hashtags after using the Instagram and TikTok search functions to preliminarily identify the most popular hashtags among alcohol‐related posts. To collect Instagram data, we used the Apify‐hosted Instagram Hashtag Scraper, which uses Instagram's internal GraphQL endpoints to retrieve posts made by public profiles or with publicly available URLs (Apify, 2021). Similarly, we used the TikTok Scraper to collect our TikTok data, which operates via TikTok's hashtag feed endpoints to retrieve public content (Clockworks, 2021). Both scrapers extract post metadata, including URLs, account usernames, captions, and shares, and export data as CSV files.

We collected all posts on May 12, 2024. Our search returned 83,285 Instagram posts and 3622 TikTok posts made on or before our collection date, from which we chose a 2% random subsample of collected Instagram posts (*n* = 1666) and a 20% random subsample of collected TikTok posts (*n* = 725). Our random subsampling strategy ensured human coding feasibility, and we based our selected percentages on prior research using large social media datasets (Gao et al., 2020; Sidani et al., 2020; Southwick et al., 2021). We then imported our Instagram and TikTok samples into Microsoft Excel spreadsheets containing a link to each post and columns with numeric identifiers and coding categories. The University of Pittsburgh Institutional Review Board did not consider this study human subjects research. Nonetheless, to minimize ethical concerns associated with user privacy, we reference notable posts as generally as possible and have omitted account usernames throughout our reporting.

### Codebook

We simultaneously developed codebooks for the coding of Instagram and TikTok posts. Our codebooks were iteratively informed by the focus of our research question, an inductive preliminary analysis of alcohol‐related social media posts, and previous analyses of social media content related to nicotine and tobacco products (Sidani et al., 2019, 2020, 2022). Each codebook included identical coding categories with minor definitional updates given differences in content format on Instagram and TikTok (i.e., images on Instagram and videos on TikTok).

Our codebook first included a series of codes assessing the availability and language of each post (English or not). If coders identified a post as unavailable, age‐restricted, or lacking context to determine the content of a non‐English language post, they were not to analyze it further. Then, our codebook included a binary code for coders to assess the relevance of each post. We operationalized posts as *relevant* if they were about alcohol use or alcohol products in the context of alcohol for human consumption (i.e., drinking). *Irrelevant* posts, for example, included those referencing art made with alcohol‐based ink or barbers spraying alcohol after a haircut.

We coded relevant posts for multiple categories (see Table S1, for codebook definitions and example posts). Codes included country of origin (i.e., the *United States* or *any other country*) and author type. Mutually exclusive options included *news* organizations, *academic or medical institutions*, *government* accounts, *commercial or marketing*‐related accounts, an individual *person* or a couple, or a *group* that was not associated with any other code‐specific organizations (e.g., a “Beer Mile” club). We also included codes for posts made by an *influencer*, defined as an individual with at least 10,000 followers, posts made by an individual or group presenting as a *medical professional*, and posts tagged as *sponsored or promoted content* made by individuals or groups collaborating with any alcohol product‐related commercial organization.

We included codes for the featured alcohol product in each post including *wine*, *beer*, alcoholic *cider*, a *flavored malt beverage*, a *hard seltzer*, a *cocktail* (i.e., any time liquor was mixed with a nonalcoholic beverage), *liquor*, *nonalcoholic* products (e.g., nonalcoholic or alcohol‐free beer or mocktails), or an *unknown product*. Mutually exclusive codes assessed the sentiment and primary purpose of each post. Sentiment codes included *pro‐alcohol*, *anti‐alcohol*, *neutral/unbiased*, *nuanced*, and *unknown*. Purpose codes included *informational*, *comedic*, *product*, *documentary*, *asking for information*, and *other*. To account for differences in content format between the Instagram and TikTok platforms, we expanded the definitions for several of the Instagram codebook purpose codes to include additional guidance. The *comedic* purpose code included posts that did not necessarily show alcohol in the picture (e.g., a text post describing Wednesdays as a “special occasion” for wine), the *product* code specified that posts should generally include a product name or label, and the *documentary* code included still image “slice of life” posts featuring someone with an alcohol product with little to no commentary (e.g., an image of someone holding a glass of wine with no other description).

A series of content codes assessed post characteristics. These included codes for posts with an explicit mention of the link between alcohol and *cancer* risk, posts explicitly showing or mentioning being drunk, binge drinking, or high‐intensity drinking (*intoxication; binge or high intensity drinking*), posts explicitly mentioning *sobriety* (i.e., total abstinence from alcohol use), and posts explicitly mentioning mindful alcohol use or the reduction of one's alcohol intake (i.e., the “*sober curious*” movement). Finally, we included a series of codes indicating posts targeting several groups including *young people* (i.e., people under the age of 21 or college‐aged), “*wine moms*” (i.e., mothers portrayed as using alcoholic beverages to cope with parenting‐related stress or fatigue), *racial or ethnic minorities*, and *sexual or gender minorities*.

### Coding procedures

Our coding process began with the independent double‐coding of training‐specific random samples of 450 Instagram posts and 400 TikTok posts. During this training period, we added, collapsed, and iteratively updated codebook definitions based on feedback from our two experienced coders and adjudications by senior members of the research team. We used Cohen's *κ* and percent agreement scores to assess interrater agreement (Cohen, 1960). After seven training rounds of Instagram posts, final *κ* coefficients reached 1.0 for post availability, 0.88 for language, and 0.92 for relevance, with all other codes reaching acceptable levels ranging from 0.65 to 1.00. After eight rounds of TikTok posts, final *κ* coefficients reached 1.0 for post availability, 1.0 for language, and 1.0 for relevance, with all other codes reaching acceptable levels ranging from 0.61 to 1.00 (Landis & Koch, 1977; Neuendorf, 2017). For codes with unreliable kappa scores given low sample prevalence, we used an acceptability threshold of 95% percent agreement between our two coders. Percent agreement for codes assessed this way ranged from 96.0% to 100% for both Instagram and TikTok posts. Complete details of our interrater reliability measures, including uncertainty surrounding our *κ* coefficient estimates, are in Table S1. Once our coders completed training for both Instagram and TikTok posts, we finalized our codebooks and the two coders each began the independent single‐coding of approximately half of our analytical samples.

### Analysis

**FIGURE 1:**
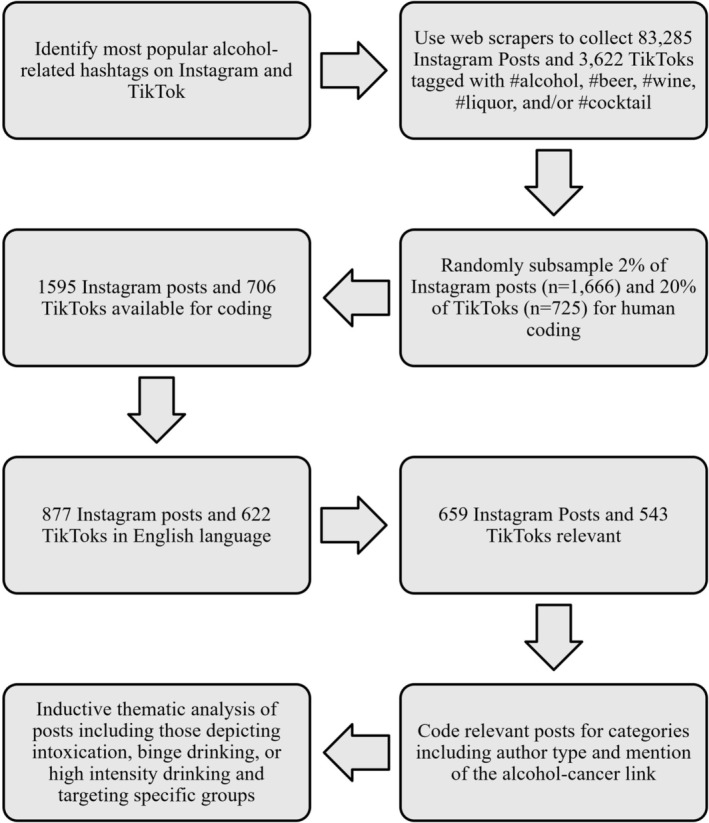
Data collection and content analysis process.

We calculated frequencies and percentages of each code for relevant Instagram and TikTok posts. Then, for a more comprehensive understanding of the alcohol‐related social media landscape, we qualitatively explored posts coded with *drinking*, *influencer*, *product*, and as targeting *young people*, *wine moms*, and *racial or ethnic minorities* using an inductive thematic content analysis (Braun & Clarke, 2006). During this iterative process, research team members reviewed coded Instagram and TikTok posts over several rounds, noted potential themes present within codes, met to discuss and reach consensus on identified themes, and developed a list of identified themes upon reaching thematic saturation (i.e., when additional posts no longer yielded novel concepts or meaningfully expanded existing themes). For frequently appearing codes, we purposefully selected posts made by different authors based on their account usernames. We omitted Instagram posts coded as *drinking*, targeting *young people*, and targeting *racial or ethnic minorities*, as well as Instagram posts and TikToks targeting *sexual or gender minorities* from our thematic analysis due to having fewer than 10 posts per category. Figure 1 provides an overview of our data collection and content analysis process.

## RESULTS

Coders identified 659 (39.6%) of posts in our Instagram random subsample and 543 (74.9%) of posts in our TikTok random subsample as relevant. 5.8% of relevant Instagram posts were tagged with #alcohol, compared to 37.6% with #beer, 45.1% with #wine, 11.7% with #liquor, and 8.3% with #cocktail. Among relevant TikTok posts, 25.6% were tagged with #alcohol, compared to 22.5% with #beer, 19.5% with #wine, 25.6% with #liquor, and 4.1% with #cocktail. Table 1 presents the frequencies and percentages for each code by social media platform for relevant posts. Across both Instagram and TikTok samples, only one post, a TikTok, mentioned the link between alcohol consumption and increased cancer risk. In this video, tagged with #alcohol, someone presenting themselves as a medical student cited increasing colon cancer incidence among young adults as a reason for alcohol abstinence among the population.

**TABLE 1:** Descriptive statistics for relevant posts by social media platform, number (%).

	Instagram (*n* = 659)	TikTok (*n* = 543)
United States	269 (40.8)	299 (55.1)
Author type^a^		
News	1 (0.2)	3 (0.6)
Academic or medical institution	0 (0.0)	0 (0.0)
Government	0 (0.0)	0 (0.0)
Commercial or marketing	320 (48.6)	39 (7.2)
Person	306 (46.4)	475 (87.5)
Group	32 (4.9)	26 (4.8)
Influencer	13 (2.0)	370 (68.1)
Medical professional	0 (0.0)	2 (0.4)
Sponsored or promoted content	1 (0.2)	1 (0.2)
Featured alcohol product^b^		
Wine	313 (47.5)	128 (23.6)
Beer	248 (37.6)	157 (28.9)
Cider	1 (0.2)	0 (0.0)
Flavored malt beverage	0 (0.0)	4 (0.7)
Hard seltzer	2 (0.3)	15 (2.8)
Cocktail	66 (10.0)	87 (16.0)
Liquor	58 (8.8)	172 (31.7)
Nonalcoholic product	3 (0.5)	8 (1.5)
Unknown product	7 (1.1)	62 (11.4)
Sentiment^a^		
Pro‐alcohol	606 (92.0)	431 (79.4)
Anti‐alcohol	1 (0.2)	21 (3.9)
Neutral or unbiased	3 (0.5)	9 (1.7)
Nuanced	5 (0.8)	65 (12.0)
Unknown	44 (6.7)	17 (3.1)
Purpose^a^		
Informational	59 (9.0)	131 (24.1)
Comedic	12 (1.8)	286 (52.7)
Product	459 (69.7)	77 (14.2)
Documentary	127 (19.3)	42 (7.7)
Asking for information	0 (0.0)	1 (0.2)
Other	2 (0.3)	6 (1.1)
Content^b^		
Cancer	0 (0.0)	1 (0.2)
Intoxication; binge or high intensity drinking	4 (0.6)	134 (24.7)
Sobriety	1 (0.2)	4 (0.7)
Sober curious	0 (0.0)	4 (0.7)
Targeting^b^		
Young people	1 (0.2)	23 (4.2)
Wine moms	35 (5.3)	10 (1.8)
Racial or ethnic minorities	2 (0.3)	24 (4.4)
Sexual or gender minorities	3 (0.5)	5 (0.9)

^a^ Author type, sentiment, and purpose categories are mutually exclusive.

^b^ Featured alcohol product, content, and targeting categories are not mutually exclusive and their proportions will not always add up to 100%.

Coders identified a minority of Instagram posts as being from the United States (40.8%), compared to over half of TikToks (55.1%). Commercial or marketing entities made 48.6% of Instagram posts, with 46.4% posted by individual accounts, and coders found that influencers made only 2.0% of Instagram posts. Individuals frequently posted TikToks (87.5%) with 68.1% having been made by someone identified as an influencer. Many Instagram posts featured wine (47.5%) or beer (37.6%), whereas 23.6% of coded TikToks featured wine, 28.9% featured beer, 16.0% featured cocktails, and 31.7% featured liquor.

A positive sentiment toward alcohol use was common across both platforms, with 92.0% of Instagram and 79.4% of TikTok posts coded as pro‐alcohol. 12.0% of TikToks also portrayed alcohol with both positive and negative sentiments. The primary purpose of 69.7% of Instagram posts was to show an alcohol product, and 5.3% of posts targeted wine moms. Comedic posts made up a majority of TikToks (52.7%) and 24.7% featured intoxication, binge drinking, or high‐intensity drinking. Our coders observed small proportions of TikTok posts targeting young people (4.2%), wine moms (1.8%), racial or ethnic minorities (4.4%), and sexual or gender minorities (0.9%).

Our qualitative thematic analysis revealed that across both platforms, people often consumed beverages containing large amounts of alcohol (e.g., a glass of wine able to hold the content of an entire bottle or a cocktail containing over seven standard drinks) quickly and as single drinks. Among the 13 Instagram posts coded as *influencer*, identified themes included the use of artistic or aesthetically pleasing photographs, along with many influencers who appeared to be representing craft breweries, vineyards, or winemakers. *Influencer*‐posted TikToks, mostly meant to be humorous, were often posted by self‐identified lifestyle, “mommy,” or alcohol micro‐influencers (i.e., influencers with relatively small followings). Additionally, small‐scale, seemingly independent commercial organizations (e.g., liquor stores, craft breweries, or wineries) primarily made Instagram and TikTok posts showing alcohol *products*. These posts often provided advertisement‐like information about luxury goods, such as celebrity‐endorsed liquors or expensive wines, or brand‐specific recommendations for cheaper but ABV or quality‐equivalent beverages. Instagram and TikTok posts identified as targeting *wine moms*, frequently made by alcohol outlets or micro‐influencers, promoted wine as a gift for Mother's Day or as closely associated with motherhood. TikToks coded as targeting *young people* incorporated social media trends and conceptualized drinking as part of the college experience, while those coded as targeting *racial or ethnic minorities* frequently included music associated with various groups (e.g., Latin music playing in posts targeting individuals with Hispanic or Latino identities). Finally, themes identified among TikToks coded as *intoxication; binge or high intensity drinking* included the frequent use of humor, generally via skits reenacting intoxication and making light of alcohol‐related consequences, and videos of people chugging multiple standard drinks at once.

### Post hoc exploratory analysis

After completing our systematic content analysis, we searched for “cancer” in the complete metadata of our total Instagram and TikTok samples. In our Instagram sample, we found that 51 of the 83,285 posts mentioned “cancer” in some capacity. Most of the relevant posts were about cancer awareness‐related fundraising events held at breweries or wineries. One Instagram post that was not included in our randomly selected subsample described the association between alcohol consumption and breast cancer and advertised regular “alcohol health testing” to monitor for prediagnosis cancer indicators. In our TikTok sample, we found that none of the three posts including “cancer” in their metadata, out of the 3622 total, described the relationship between alcohol and increased cancer risk.

## DISCUSSION

Our exploration of alcohol‐related Instagram and TikTok posts found virtually no mention of the link between alcohol and increased cancer risk. Only a single TikTok, representing <1% of coded posts, mentioned the association between alcohol consumption and increased cancer risk. Posts largely portrayed drinking with a positive sentiment: Over 90% of Instagram and almost 80% of TikTok posts presented desirable aspects of alcohol use with little to no mention of negative consequences. Our thematic analysis further revealed that in our sample what constituted a single drink was often well beyond current United States daily guidelines for alcohol consumption, with beverages containing large amounts of alcohol consumed quickly and often with comedic intent, particularly on TikTok.

Our findings as to the lack of messaging about the alcohol‐cancer link and the general content presented in alcohol‐related social media posts are consistent with previous literature. For example, work examining the prevalence of alcohol‐cancer information among Twitter posts using cancer‐related hashtags found that while risk communication was common among posts mentioning alcohol, such posts were exceedingly rare and infrequently recommended limiting alcohol use (King et al., 2023). The absence of cancer risk communication may be especially problematic in the context of alcohol‐related content, since social media users viewing or engaging with alcohol‐related content are more likely to drink and thus at higher risk for developing alcohol‐associated cancers (Curtis et al., 2018). Prior research has also identified a high prevalence of TikTok posts featuring positive portrayals of alcohol use, along with content featuring the chugging of high‐alcohol content beverages (Clement et al., 2025; Russell et al., 2021). Exacerbated by the positive sentiment surrounding alcohol use, pervasive hazardous drinking on social media may contribute to platform users' attitudes and behaviors, potentially normalizing excessive alcohol use and increasing risks for alcohol‐related harms including cancer.

We found that content posted by individual influencers with small followings or commercial entities made up most of the posts in our sample, while news organizations, academic or medical institutions, and government entities had little to no presence. Influencers posting alcohol‐related content often self‐identified as niche lifestyle, alcohol, or motherhood‐related content creators. Much of their content was meant to be comedic in nature, and regularly featured intoxication, binge, or high intensity drinking, consistent with depictions of alcohol intoxication on other social media platforms, such as YouTube (Primack et al., 2015). Posts made by small‐scale commercial or marketing organizations (e.g., craft breweries, wineries, and liquor stores) reflected the common use of social media as an alcohol marketing space, though were rarely tagged as sponsored or promoted content (Carah & Brodmerkel, 2021; Crocetti et al., 2025; Lobstein et al., 2017). Associated with increased alcohol consumption, the high prevalence of alcohol marketing on social media, coupled with the lack of messaging from public health or broader scientific perspectives, may present an additional challenge to reducing alcohol‐associated cancer incidence (Giesbrecht et al., 2024).

Shifting alcohol consumption patterns, particularly among women in their 30s and 40s, highlight specific groups that would most benefit from targeted social media messaging about alcohol and cancer (Keyes et al., 2019). Heavily exposed to messaging about the supposed health benefits of low‐to‐moderate alcohol consumption, women in this age group may also engage most frequently with wine mom content on social media given overlap with common parenting ages. Wine mom culture has become widespread on social media and frequently encourages alcohol consumption as a “healthy” part of motherhood (Basch et al., 2021; Hill & Mazurek, 2024). We found that alcohol‐related posts targeting wine moms commonly promoted wine as a Mother's Day gift or as otherwise closely linked to motherhood, consistent with previously identified themes of wine as a commodity tied to social belonging among mothers (Harding et al., 2021). Similarly, posts targeted toward young people frequently included popular social media trends, reflecting strategies for the delivery of alcohol‐related content to specific, potentially susceptible populations.

Platform‐specific features and algorithms may exacerbate challenges posed by the normalization of alcohol misuse, rampant marketing, and subgroup targeting with important implications for cancer risk communication. TikTok's “For You Page,” for example, prioritizes content that rapidly accumulates engagement and capitalizes on trending audio and visual formats, which could broadly amplify alcohol‐related posts that portray hazardous drinking in entertaining or socially desirable ways (Zhang & Liu, 2021). In contrast, Instagram's “Feed” emphasizes content shared within users' existing networks, reinforcing product promotion and associated peer‐based drinking norms (Mosseri, 2023). United States‐based public health communication seeking to tap into the alcohol‐related content landscape must consider how platforms themselves shape content creation and proliferation and can leverage platform attributes to advance public awareness of the alcohol‐cancer link. International campaigns have provided evidence for platform‐aware features likely important for cancer risk communication on social media (Booth et al., 2023; Christensen et al., 2019; Martin et al., 2018). Focused on succinctly conveying cancer risk as a reason to change drinking behavior and featuring evocative, engagement‐driving imagery, such as spilled wine slowly spreading to depict where alcohol‐attributable cancers occur in the body, these and other campaigns have incorporated features associated with both increased motivation to reduce drinking and social media relevance (Wakefield et al., 2017). Perhaps especially well‐suited for rapidly emerging short‐form social media content, developing similar messaging for an American audience should be a priority.

### Limitations

This study has several limitations. First, our findings are descriptive only, as we did not conduct statistical tests comparing Instagram and TikTok posts. Alcohol‐related content on Instagram and TikTok may also not be comparable to that found across all social media platforms and may only reach people seeking alcohol‐related posts. Nonetheless, we offer insight into an important content area relevant to alcohol use and public awareness of the alcohol‐cancer link. Second, our data collection approach captured a snapshot of what someone might encounter if searching for alcohol‐related hashtags among public posts on a given day. While our sample contained posts made on or before May 12, 2024, the exact date of posting was not recorded as it was not pertinent to our research question, and we may be missing other hashtags associated with discussion of the alcohol‐cancer link on social media. Future research should select scraping tools that return the date of posting should it be relevant to their study and include posts with both alcohol and cancer‐related hashtags, which may be more likely to include messaging about the relationship between alcohol and cancer risk. Third, we chose to focus our study on the explicit mention of cancer to avoid conflating cancer risk communication with broader, and potentially more prevalent, narratives referencing other alcohol‐associated health risks, such as liver disease and alcohol‐attributable injury. This approach may exclude subtler framings or implicit references to cancer, which we view as an importance avenue for later research. Finally, interrater reliability measures for several of our coding categories met only moderate levels of agreement (i.e., *κ* coefficients between 0.6 and 0.8), around which there is substantial ongoing debate, especially in the context of interpreting social media posts (Neuendorf, 2017). To mitigate these concerns and ensure the rigor of our approach, we followed a systematic and validated coding procedure throughout our exploratory analysis. Nonetheless, our results should be interpreted with caution given uncertainty around many of our *κ* coefficient estimates.

### Future directions

Future studies can use our results to inform broader content analyses of social media posts on Instagram, TikTok, and other popular platforms, including additional hashtag search terms to describe wider swathes of the alcohol‐cancer social media landscape. Potential searches could include posts tagged with both cancer and alcohol‐related terms and focus on identifying the attributes of such content with the widest reach and highest engagement (e.g., comments, likes, and shares). These subsequent analyses, supplemented with evidence from prior international campaigns, can inform the development of social media content about the alcohol‐cancer link and potentially reveal opportunities for collaborative messaging development with successful post authors (Booth et al., 2023; Christensen et al., 2019; Martin et al., 2018). Additionally, given the issuance of the Surgeon General's Advisory on January 3, 2025, about the link between alcohol and increased cancer risk, released after our data collection period, future studies can assess longitudinal changes in social media communication about the relationship between alcohol and cancer risk before and after the release of the advisory (Office of the Surgeon General, 2025).

## CONCLUSION

The prevalence of alcohol‐cancer risk communication among alcohol‐related social media posts is low. Instagram and TikTok content frequently portray alcohol use with a positive sentiment and depict beverages containing large amounts of alcohol as single drinks. Content analyses can provide insight into tailoring platform‐aware social media messaging to increase public awareness of the alcohol‐cancer link among American adults.

## CONFLICT OF INTEREST STATEMENT

The authors declare no known conflicts of interest.

## Supporting information


Table S1.

## Data Availability

The data that support the findings of this study are available from the corresponding author upon reasonable request.
