# Supplementary material for: Alcohol‐cancer risk communication on social media: A content analysis of alcohol‐related Instagram and TikTok posts
Source: Alcohol Clin Exp Res (Hoboken). 2026 Feb 22;50(2):e70260. doi: 10.1111/acer.70260 (PMC12926520; doi:10.1111/acer.70260)
Supplement: Supplementary file 1 — Table S1. [file ACER-50-0-s001.docx]

| \| Table S1. Definitions for Instagram and TikTok codes, measures of inter-rater reliability, and example post content. \| \| --- \| | | | | |
| --- | --- | --- | --- | --- | --- |
| Code |  |  |  |  |
| *Sub-code* | **Instagram Cohen’s κ (95% CI) or Percent Agreement** | **TikTok Cohen’s κ (95% CI) or Percent Agreement** | **Definition** | **Example Content** |
| United States | 1.00 (0.72, 1.00) | 0.81 (0.53, 1.00) | The post mentions a US location in their bio or other posts by the account mainly show the US. | - Image post by an Orlando, Florida liquor store. - Video referencing the American Midwest. |
| Author Type* |  |  |  |  |
| *News* | 96.0% | 1.00 (0.72, 1.00) | The post is authored by a news organization. | - Image post by a news outlet referencing a beer shortage. - Video posted by a news outlet about the process of making mezcal. |
| *Academic or Medical Institution* | 100%** | 1.00 (0.72, 1.00) | The post is authored by an academic or medical institution account. | - None in Instagram analytical sample. - None in TikTok analytical sample. |
| *Government* | 100%** | 100%** | The post is authored by a government account. | - None in Instagram analytical sample. - None in TikTok analytical sample. |
| *Commercial or Marketing* | 0.77 (0.49, 1.00) | 1.00 (0.72, 1.00) | The post is authored by a supplier, distributor, or marketer of alcohol products. | - Image post by an Austrian pub. - Video posted by a liquor store. |
| *Person* | 0.95 (0.67, 1.00) | 0.85 (0.57, 1.00) | The post is authored by an individual person or a couple (i.e., an account for personal use). | - Image post by someone holding a glass of wine and a cigar. - Video posted by someone explaining her use of a recycling program to purchase a box of wine. |
| *Group* | 0.66 (0.47, 0.84) | 0.65 (0.39, 0.91) | The post is authored by an organization that is not associated with a news outlet, academic or medical institution, or alcohol product-related commercial entity. | - Image posted by an account associated with a collective of liquor reviewers. - Video posted by an account associated with a podcast. |
| Influencer | 1.00 (0.72, 1.00) | 0.88 (0.60, 1.00) | The post author is an individual with at least 10,000 followers. | - Image posted by a solo traveler account with more than 10,000 followers. - Video posted by an account with more than 10,000 followers. |
| Medical Professional | 100%** | 100%** | The post is by an individual or group presenting as a medical professional, such as a nurse, doctor, or dietician. | - None in Instagram analytical sample. - Video posted by someone describing themselves as a “neuroscientist” who helps people prevent Alzheimer’s disease. |
| Sponsored or Promoted Content | 100%** | 100%** | The post is by an individual or group explicitly collaborating with or sponsored by any commercial organization related to alcohol products. | - Imaged post tagged with a “paid partnership” indicator. - Video tagged with a “paid partnership” indicator. |
| Featured Alcohol Product |  |  |  |  |
| *Wine* | 0.95 (0.67, 1.00) | 0.90 (0.62, 1.00) | The post features wine. | - Image post of someone holding a glass of wine. - Video showing how to get the last remaining wine out of a box. |
| *Beer* | 1.00 (0.72, 1.00) | 1.00 (0.72, 1.00) | The post features beer. | - Image post of a half-full beer glass. - Video of someone drinking “cold ones” when bored. |
| *Cider* | 100%** | 100%** | The post features alcoholic cider. | - Image post showing mango-flavored cider on tap at a bar. - None in TikTok analytical sample. |
| *Flavored Malt Beverage* | 100%** | 1.00 (0.72, 1.00) | The post features a flavored malt beverage. | - None in Instagram analytical sample. - Video referencing a “sidewalk slammer” (i.e., Four Loko and 40-ounce malt liquor combined into a single beverage). |
| *Hard Seltzer* | 100%** | 1.00 (0.72, 1.00) | The post features alcoholic seltzer. | - Image post featuring someone holding an alcohol seltzer. - Video showing a new hard seltzer product in a liquor store. |
| *Cocktail* | 0.85 (0.57, 1.00) | 0.88 (0.60, 1.00) | The post features a cocktail. | - Image post showing a cocktail next to a full beer glass. - Video showing an espresso martini. |
| *Liquor* | 1.00 (0.72, 1.00) | 0.82 (0.55, 1.00) | The post features liquor. | - Image post showing a group of people about to drink shots of liquor. - Video of someone drinking flavored rum. |
| *Non-Alcoholic Product* | 99.0% | 98.0% | The post features a non-alcoholic or alcohol-free product. | - Image post showing an advertisement for non-alcoholic cider. - Video of someone reviewing non-alcoholic beer for consumption during pregnancy. |
| *Unknown Product* | 0.88 (0.60, 1.00) | 0.79 (0.52, 1.00) | The post features an unknown alcohol or alcohol-related product. | - Image post by a club advertising unspecified “drinks.” - Video mentioning unspecified “alcohol.” |
| Sentiment* |  |  |  |  |
| *Pro-alcohol* | 0.88 (0.60, 1.00) | 0.65 (0.38, 0.93) | Alcohol use is portrayed in a positive way. | - Image post reviewing a beer and describing it as “leaving a great first impression.” - Video of someone describing alcohol as making them “magically feel better.” |
| *Anti-alcohol* | 99.0% | 1.00 (0.72, 1.00) | Alcohol use is portrayed in a negative way. | - Image post captioned “SOBER.” - Video mentioning “throwing-up” from drinking. |
| *Neutral or Unbiased* | 100%** | 1.00 (0.72, 1.00) | The post is neutral or unbiased towards alcohol use. | - Image post of a wine bottle with an unrelated caption. - Video of someone describing the use of a fridge lock to safely store their alcohol. |
| *Nuanced* | 98.0% | 0.79 (0.52, 1.00) | Alcohol use is portrayed with both positive and negative aspects. | - Post of several images making fun of the bad taste and consequences of drinking liquor. - Video describing positive and negative stereotypes associated with different alcoholic beverages. |
| *Unknown* | 96.0% | 96.0% | The sentiment of the post towards alcohol use or alcohol products is unclear. | - Image post of a beer glass with no caption. - Video skit of someone setting a reminder to purchase alcohol at 5:00pm. |
| Purpose* |  |  |  |  |
| *Informational* | 98.0% | 0.65 (0.39, 0.91) | The primary purpose of the post is to “teach” or “explain” something. | - Image post about winemaking in Argentina. - Video of someone showing how to make a cocktail. |
| *Comedic* | 0.66 (0.40, 0.92) | 0.92 (0.64, 1.00) | The primary purpose of the post is to entertain, including making light of the consequences of alcohol. Instagram posts do not necessarily show alcohol in the picture. | - Image post of someone holding a glass of wine as the “original fruit smoothie.” - Animated video of someone dancing while drunk. |
| *Product* | 0.84 (0.56, 1.00) | 0.79 (0.51, 1.00) | The primary purpose of the post is to show an alcohol-related product, including a bar or company selling a product. Instagram posts should generally have a product name or label. | - Image post featuring liquor bottles and advertising an alcohol delivery service. - Video posted by a liquor store showcasing celebrity-associated alcohol products. |
| *Documentary* | 99.0% | 98.0% | The primary purpose of the post is to show or ponder life. Includes Instagram post of someone holding an alcoholic beverage with little commentary. | - Image and video post of someone documenting their experience at a winery. - Video describing the receipt of several bottles of homemade wine as a gift. |
| *Asking for Information* | 98.0% | 98.0% | The primary purpose of the post is to ask a question about an alcohol product. | - None in Instagram analytical sample. - Video asking commenters to guess the top-selling alcohol products at a bar. |
| *Other* | 100% | 100%** | The primary purpose of the post is something other than informational, comedic, product, documentary, or asking for information. | - Image post with a caption describing a labor strike by Canadia liquor store employees. - Video of people singing a song about beer. |
| Content |  |  |  |  |
| *Cancer* | 1.0 (0.80, 1.00) | 100%** | The post explicitly mentions the relationship between alcohol and cancer risk. | - None in Instagram analytical sample. - Video describing trends in young adult alcohol use and alcohol’s association with several types of cancer. |
| *Intoxication; Binge or High Intensity Drinking* | 100%** | 0.67 (0.40, 0.95) | The post explicitly shows or mentions being drunk, binge drinking, or high intensity drinking. | - Image post with text making fun of spring breakers getting drunk in Florida. - Video of a woman quickly drinking 4 glasses of wine. |
| *Sobriety* | 100%** | 1.00 (0.72, 1.00) | The post explicitly mentions sobriety or total abstinence from alcohol use. | - Image of someone sitting in a bar or restaurant captioned “SOBER.” - Sliding image post describing dangers of alcohol and need for alcohol abstinence. |
| *Sober Curious* | 99.0% | 98.0% | The post explicitly mentions mindful alcohol use or reducing alcohol intake, including mention of “Dry January” or “Sober October.” | - None in Instagram analytical sample. - Video positively depicting Sunday morning after choosing not to go out drinking the night before. |
| Targeting |  |  |  |  |
| *Young People* | 98.0% | 98.0% | At least one aspect of the post targets people college-aged or under the age of 21. | - Image post featuring college-aged people drinking at a party. - Video showing German university students opening beer during a university lecture. |
| *Wine Moms* | 1.00 | 98.0% | At least one aspect of the post targets “wine moms” (i.e., references wine and motherhood). | - Image post advertising Mother’s Day specials at a wine store. - Video of a mom dropping off her children and receiving a bottle of wine in exchange. |
| *Racial or Ethnic Minorities* | 100%** | 0.66 (0.40, 0.92) | At least one aspect of the post targets racial or ethnic minorities. | - Image post of a tasting event held by a Black-owned alcoholic beverage company. - Video of someone making a drink with Latin music playing in the background. |
| *Sexual or Gender Minorities* | 99.0% | 98.0% | At least one aspect of the post targets sexual or gender minorities | - Image post of someone drinking wine in a park tagged with #gay and #instagay. - Video tagged with #lgbt and #lgbtq. |

*Author Type, Sentiment, and Purpose categories are mutually exclusive. **Indicates codes that did not affirmatively appear in our Instagram or TikTok coding training samples.
